# The melectine bee genera *Brachymelecta* and *Sinomelecta* (Hymenoptera, Apidae)

**DOI:** 10.3897/zookeys.244.3979

**Published:** 2012-11-19

**Authors:** Michael S. Engel, Charles D. Michener

**Affiliations:** 1 Division of Entomology, Natural History Museum, and Department of Ecology & Evolutionary Biology, 1501 Crestline Drive – Suite 140, University of Kansas, Lawrence, Kansas 66045, USA

**Keywords:** Apoidea, Anthophila, Apinae, Melectini, Nevada, China, taxonomy, morphology

## Abstract

The enigmatic, cleptoparasitic bee genera *Brachymelecta* Linsley and *Sinomelecta* Baker (Apinae: Melectini) are redescribed, each represented by a single species which has not been reencountered since capture of the type series ca. 1878 and 1900, respectively. Both genera are the only melectines to possess two submarginal cells in the forewing but are otherwise wholly dissimilar. *Brachymelecta mucida* (Cresson), a species known only from the male holotype collected in “Nevada”, is newly described and figured, including the first account of the hidden sterna and genitalia. *Sinomelecta oreina* Baker is similarly described and figured based on the holotype male and paratype female, apparently collected from the eastern Tibetan Plateau. Both genera are valid and from the available data do not appear to represent merely autapomorphic forms of *Melecta* Latreille. Indeed, the terminalia of *Sinomelecta oreina* are in some respects more similar to those of species of *Thyreus* Panzer.

## Introduction

The objective of this paper is to fully describe two genera of the anthophorine tribe Melectini. Each is known from only one or two specimens collected over a century ago and preserved without adequate locality data. One or both may now be extinct. The two genera do not appear to be close relatives, although they share a striking common character: both have only two submarginal cells in the forewing although all other Melectini have three. The genera involved are *Brachymelecta* Linsley and *Sinomelecta* Baker; each is discussed and described below. Since each genus contains only a single species, the descriptions combine specific characters with probable generic characters, i.e., characters that differentiate other genera of Melectini. A very unusual feature of *Sinomelecta* is that the male, like the female, has 12 antennomeres. In nearly all bees males have 13 antennomeres. There is no reason to believe that *Brachymelecta* shares this character but since the antennae of the sole specimen of that genus are broken, new material will be necessary if the antennomeres of that taxon are to be counted.

**Figure 1.**
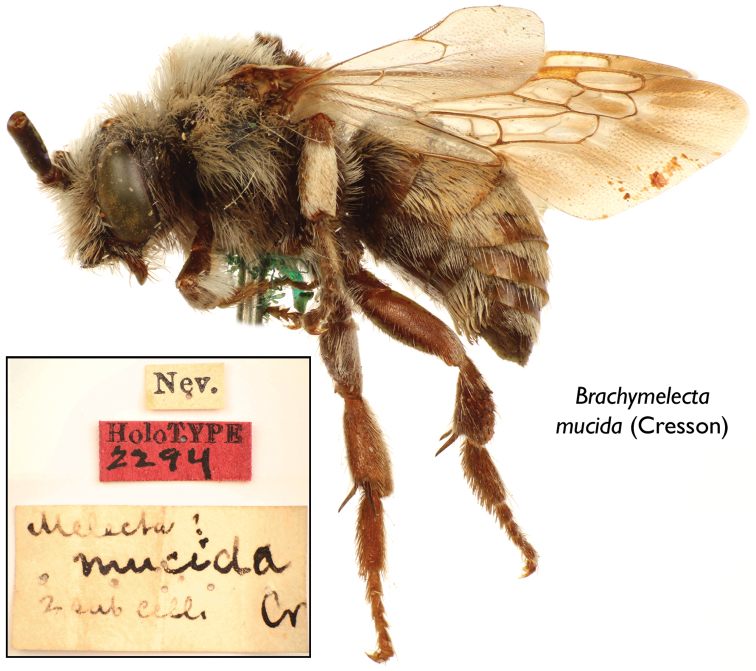
Lateral habitus of male holotype of *Brachymelecta mucida* (Cresson) (ANSP Type No. 2294); inset depicts the three original labels associated with the specimen.

Given the rarity of this material, the few references to them in the literature, and thereby their general unfamiliarity to many, it is worthwhile to summarize the historical details for each genus. The only known specimen of *Brachymelecta* is preserved in the Academy of Natural Sciences of Philadelphia. Described in 1879 as “*Melecta*? *mucida*”, its source was given as “Nevada (Morrison)” (Cresson 1879a) although the label on the specimen is merely “Nev.” (Fig. 1). The label (“Nev.”) on the specimen is exactly like labels on other Hymenoptera described by Cresson that do occur in Nevada and adjacent states. Of course an error in labeling is possible but there is no evidence of such an error. Cresson described various Hymenoptera collected by Morrison, sometimes given as H.K. Morrison [Herbert Knowles Morrison (1854–1885)], mostly from the western United States but some from the southeast (Georgia). In spite of extensive collecting in the western United States by persons interested in bees, no other specimens have been obtained. *Brachymelecta* might be extinct, or may exist in or near Nevada, less likely elsewhere. If not extinct, it is presumably exceedingly rare. Like other Melectini, it would be a cleptoparasite in nests of other bees. The only equally small North American melectine is *Zacosmia* Ashmead which also occurs in the western United States and is a cleptoparasite of *Anthophora* (*Heliophila*).

The two known specimens of *Sinomelecta*, one of each sex, were in the collection of Donald B. Baker (1922–2004) for many years and are today preserved in the Division of Entomology of the University of Kansas Natural History Museum. They were described by Baker as *Sinomelecta oreina* in 1997 (Baker 1997). These specimens were found by Baker in a mixed batch of “dealer material” probably collected near the “turn of the century”, i.e., about 1900. With other insects of various orders, they stood above a penciled label “Sungpan” or “Songpan”. After discussing the probable meaning of this label and noting that there is no certainty that all the insects came from the same locality, Baker (1997) summarized by indicating the probability “that the *Sinomelecta* came from a montane locality on the eastern fringe of the Tibetan plateau. The general locality would approximate to the Ta-hsuëh Shan and Chiunghsia Shan of the *Times Atlas*.” That additional specimens have not been taken, so far as we know, may merely reflect the scarcity of bee collectors in this region.

## Material and methods

The material discussed herein consists of the unique type material for *Brachymelecta mucida*, housed in the Department of Entomology, Academy of Natural Sciences, Philadelphia (ANSP), while the holotype male and paratype female of *Sinomelecta oreina* are in the Division of Entomology, University of Kansas Natural History Museum, Lawrence (SEMC). Through the kindness of Jason Weitraub (ANSP) we were permitted to dissect the metasomal apex of the *Brachymelecta mucida* holotype and to clean the genitalia for imaging. Despite being over 134 years old (by comparison to the merely 112+ year old genitalia of *Sinomelecta oreina*), the genital capsule and hidden sterna were easily removed from between TVII and SVI and after a gentle bath in a weak potassium hydroxide solution, most of the surrounding connective tissue proved no challenge to detach from the sclerites. The terminalia of *Sinomelecta oreina* had been dissected and partially cleaned by the late Donald B. Baker at some point prior to his published account of the species (Baker 1997). Given the uniqueness of the material we did not subject it to further preparation and cleaning. Photomicrography was undertaken with a Nikon D1x digital camera attached to an Infinity K-2 long-distance microscope lens. Measurements were made with an ocular micrometer on an Olympus SZX-12 stereomicroscope. The following abbreviations are used in the descriptions: F, flagellomere; S, metasomal sternum; T, metasomal tergum. Morphological terminology generally follows that of Engel (2001) and Michener (2007). Given that each genus is monobasic we have composed generic diagnoses from those characters which elsewhere serve to differentiate genera in the tribe.

## Systematics

### Genus Brachymelecta

Linsley

http://species-id.net/wiki/Brachymelecta

Brachymelecta Linsley, 1939: 458. Type species: *Melecta mucida* Cresson, 1879a, by original designation. Michener 1944: 287; Michener 2000: 748, 750; Michener 2007: 771, 773.

#### Diagnosis (male).

Antenna with F1 over 1.5 times as long as F2. Mesoscutellum with subhorizontal dorsal surface about twice as long as vertical surface; dorsal and posterior surfaces divided by longitudinal depression resulting in bilobed form, posterior dorsal part of each lobe forming narrowly rounded, obtuse angle projecting posteriorly. Arolia present. Forewing with two submarginal cells (i.e., 1rs-m absent). Metasomal T1 to T4 densely covered with pale brown, appressed, plumose setae; T1 with midlength of horizontal surface subequal to that of vertical (anterior) surface and considerably shorter than midlength of exposed part of T2.

### Brachymelecta mucida

(Cresson)

http://species-id.net/wiki/Brachymelecta_mucida

Figs 1 [Fig F2] [Fig F3] –10

Melecta? *mucida* Cresson, 1879a: 205 [♂]. Cresson 1887: 298 [checklist]; Fox 1893: 143 [♂, key]; Cresson 1916: 125 [type catalog, note on broken antennae].
[Melecta]? *mucida* Cresson; Cresson 1879b: 218 [checklist].
Melecta mucida Cresson; Dalla Torre 1896: 317 [checklist].
Brachymelecta mucida (Cresson); Linsley 1939: 459 [♂].
Brachymelecta mucida (Cresson); Hurd 1953: 37 [♂, note].

#### Holotype.

♂ (Fig. 1), labeled “Nev. [presumed abbreviation for Nevada, USA]” // “Melecta? mucida, 2 sub cells, Cr” // “Holotype 2294 [red label]” (Fig. 1); deposited in the Department of Entomology, Academy of Natural Sciences, Philadelphia, Pennsylvania, USA.

#### Diagnosis.

As for the genus (*vide supra*).

#### Description.

*Male (holotype)*: Body length 9 mm, forewing length 8 mm. Head width 2.7 mm; head length (lower margin of clypeus to vertex in facial view) 2.1 mm. Intertegular distance 2.0 mm; distance between outer margins of tegulae 3.0 mm.

Clypeus strongly protuberant, in lateral view extending anteriorly about compound eye width in front of lower compound eye margin; lower margin straight, middle third slightly depressed. Mandible with distal half almost parallel sided, less than half as wide as base; apex bidentate, upper tooth slightly smaller and shorter than lower tooth; basal tooth not evident but mandibles closed and not fully exposed. Malar space very short, base of mandible closely approaching compound eye. Labrum not fully exposed but apparently about as long as broad. Inner orbits converging below (Fig. 3); vertex rather strongly and uniformly convex (Fig. 3). Gena broadest at upper third, not as broad as compound eye; preoccipital ridge sharply angulate; median ocellus with transverse diameter (= ocellar diameter) greater than that of lateral ocellus, ocellocular distance approximately equal to interocellar distance, ocelloccipital distance less than twice ocellar diameter, distance between lateral and median ocelli equal to diameter of lateral ocellus. Antenna with scape scarcely over twice as long as maximum width which is scarcely greater than width of flagellum (based on first four flagellomeres only); pedicel exposed as narrow ring about four times as broad as long; F1 over 1.5 times as long as F2; F2, 3, and 4 subequal, each broader than long, F2 and F3 together longer than F1 (Fig. 3) (antennae broken so that F5 and beyond cannot be described; breakage occurred long ago as indicated by Cresson 1916: 125). Mesoscutellum with subhorizontal dorsal surface about twice as long as vertical surface; dorsal and posterior surfaces divided by longitudinal depression resulting in bilobed form, posterior dorsal part of each lobe forming narrowly rounded, obtuse angle projecting posteriorly; angle between posterior and dorsal surfaces approximately orthogonal but not formed by carina; posterior surface not overhanging metanotum; lower margin of mesoscutellum above metanotum marked by strong transverse carina. Metatibia with outer surface coarsely nodulose; outer apical margins of tibiae protuberant but without conspicuous spines; mesotibial spur and outer metatibial spur about as long as tibial diameter; inner metatibial spur longer than tibial diameter; arolia well developed; pretarsal claws cleft, outer ramus slender, sharply pointed, inner ramus flattened, expanded, much shorter than outer ramus, apex approximately right angular [much as in *Xeromelecta (Melectomorpha) californica* (Cresson): *vide* Michener 2007: fig 117d]. Forewing with surface beyond venation strongly papillate (Fig. 4); basal vein strongly basad cu-a; submarginal cells two because of loss of 1rs-m; other aspects of wing venation shown in figures 4 and 5. Metasomal T1 with midlength of horizontal surface subequal to that of vertical (anterior) surface and considerably shorter than midlength of exposed part of T2; posterior margins of sterna straight, transverse, to gently concave on more posterior sterna to S5; S1with midbasal tubercle, not carinate; S7, S8, and genitalia illustrated in Figures 6–10.

Because of rather dense vestiture, surface in some areas seen only locally; following might change considerably if setae removed from certain areas: Clypeus coarsely and closely punctate medially, anterior margin with even larger and irregular punctures; most of remainder of head with dense coarse punctures with irregular smooth shiny areas between some punctures; lower half of frons, adjacent parts of paraocular area, and supraclypeal area dull with dense small punctures. Mesosoma largely coarsely punctate with punctures similar to those of center of clypeus but with more shiny ground between punctures which often separated by one-half puncture width although close in other areas; median part of mesoscutellum and especially mesoscutellar lobes with punctures even larger, leaving only a network of ridges; metanotum and propodeal triangle with punctures smaller, as close as they can be, on lateral part of triangle forming series of transverse (vertical) irregular striae. Metasomal punctures minute, mostly separated by several puncture diameters, surface between punctures largely lineolate, especially on sterna where large areas lack punctures almost completely; posterior margins of terga smooth.

Setae of head and mesosoma rather abundant, mostly two to three ocellar diameters in length, grayish white (cinereous) with brownish tints on lower parts of gena, blackish on axilla, largely white on sides of mesosoma and center of face; antennal scape and coxae with similar grayish white setae, mostly one ocellar diameter in length. Antennal pedicel with dense very short setae; flagellum asetose; legs beyond coxae largely with short, yellowish white setae, dense and yellow on under sides of tarsi; profemur with strong fringe of white setae two or more ocellar diameters in length on posterior surface; similar fringe of much shorter and less conspicuous white setae on mesofemur; outer surface of mesotibia except near base densely covered with white setae that obscure surface; protibia with similar white setae, less dense, and absent on both base and apex. Metasomal T1 to T4 densely covered with pale brown, appressed, plumose setae, except posterior margins smooth and bare, these margins narrow on T1 to T3, broader especially medially on T4; T5 and T6 with exposed parts like margins of more anterior terga but T6 with some pale brown plumose setae basally; T1 to T4 with a few long simple setae laterally, very few on T1, number increasing and more dorsal from T2 to T4; S1 to S3 with few long pale setae, large median areas on S2 and S3 asetose; S4 and S5 with fringes of long pale brown setae.

Integumental coloration black, legs and middle third of mandible dark reddish brown except tibial spurs black; metasomal sterna and posterior margins of terga dark brown; under side of antenna brownish black; tegula translucent brownish black. Wings transparent, shaded with dusky brown beyond venation of forewing, darkest near costal margin distal to marginal cell (Fig. 1), weakly darkened within distal cells; veins dusky brown, pterostigma light brown.

*Female*: Unknown.

**Figures 2–3. F2:**
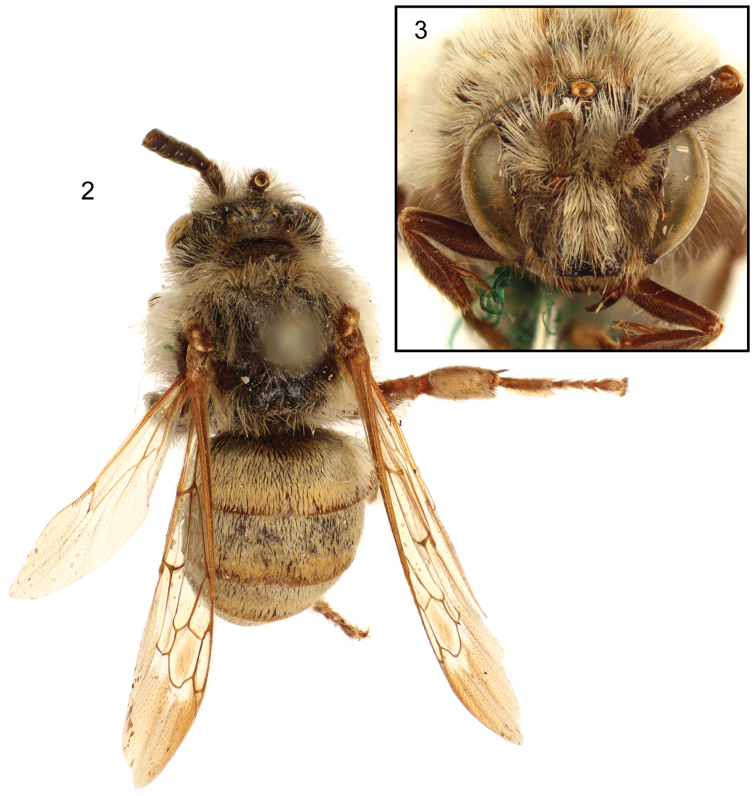
Dorsal (**2**) and facial (**3**) views of male holotype of *Brachymelecta mucida* (Cresson) (ANSP Type No. 2294).

**Figures 4–5. F3:**
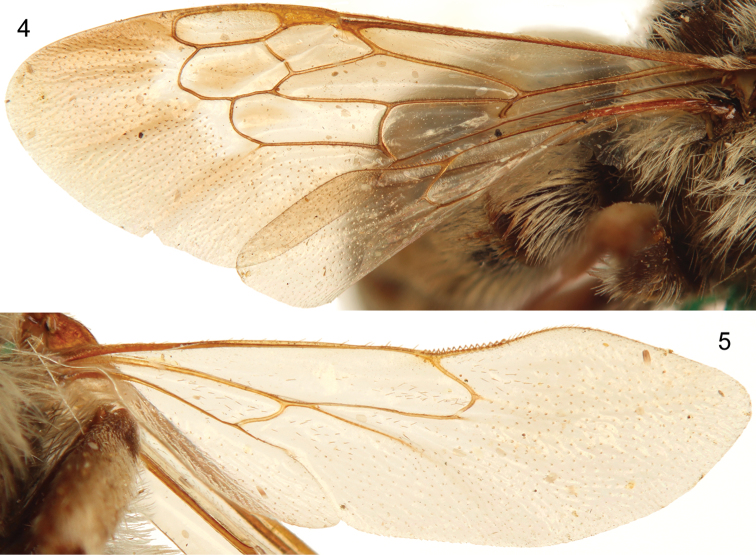
Forewing (**4**) and hind wing (**5**) of male holotype of *Brachymelecta mucida* (Cresson) (ANSP Type No. 2294).

**Figures 6–10.**
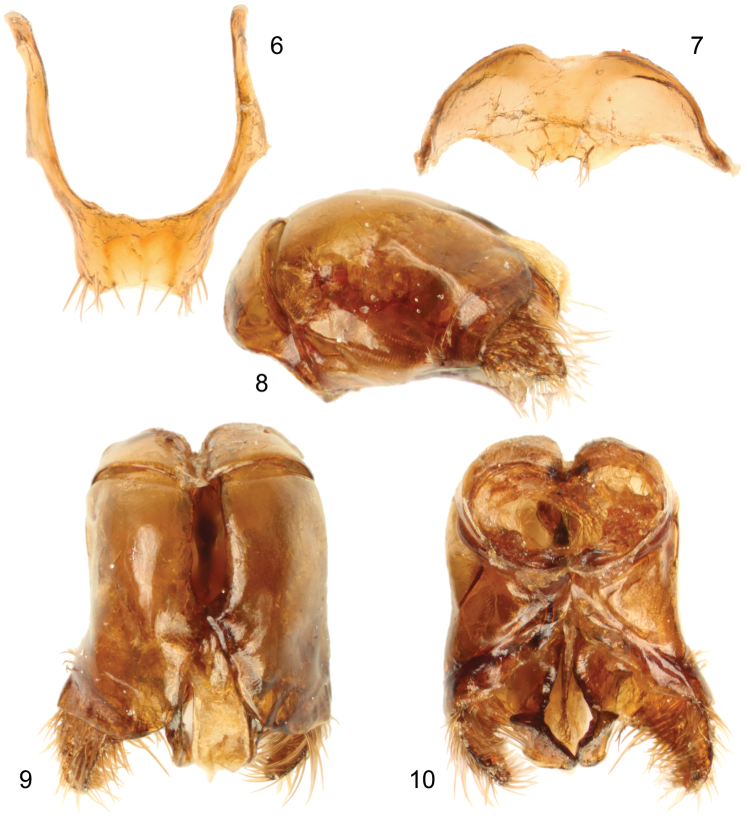
Male terminalia of holotype of *Brachymelecta mucida* (Cresson) (ANSP Type No. 2294). **6** Seventh metasomal sternum **7** Eighth metasomal sternum **8** Genital capsule, lateral view **9** Genital capsule, dorsal view **10** Genital capsule, ventral view.

#### Comments.

Cresson (1879a) records the specimen as from “Nevada, (Morrison)” (p. 205) even though the preserved label provides only “Nev.” (Fig. 1), presumably an abbreviation for Nevada. The specific epithet also appears in a checklist of North American Apidae as, “? *mucida* Cress. ibid. 205, ♂. Nevada.” (Cresson 1879b). As noted above, Cresson described various Hymenoptera collected by H.K. Morrison in Colorado, Georgia, Nevada, and elsewhere. Many of these are well known North American species. According to Mann (1885) Morrison is known to have collected in Nevada in 1878 and sold his collections back east.

### Genus Sinomelecta

Baker

http://species-id.net/wiki/Sinomelecta

Sinomelecta Baker, 1997: 245. Type species: *Sinomelecta oreina* Baker, 1997, by original designation. Michener 2000: 748, 751; Rightmyer and Engel 2003: 3, 6; Michener 2007: 771, 774.

#### Diagnosis.

Antenna with 12 antennomeres in both sexes; F1 nearly twice as long as apical width and about twice as long as F2. Body without patches of appressed, white setae. Mesoscutellum with subhorizontal dorsal surface about twice as long as subvertical surface, both surfaces divided by weak longitudinal median depression so that mesoscutellum is weakly biconvex, each convexity emphasized by small posteriorly directed sublateral projection that almost overhangs metanotum. Arolia present but small. Forewing with two submarginal cells (i.e., 1rs-m absent). Metasomal T1 with long cinereous setae laterally; T2 to T5 with similar setae, some of them brownish, at extreme sides; T2 to T4 with subapical bands of white setae (broken medially on T2); T1 with midlength of horizontal surface about half as long as declivitous anterior surface and about half as long as midlength of exposed part of T2.

### Sinomelecta oreina

Baker

http://species-id.net/wiki/Sinomelecta_oreina

**Figures 11–12.**
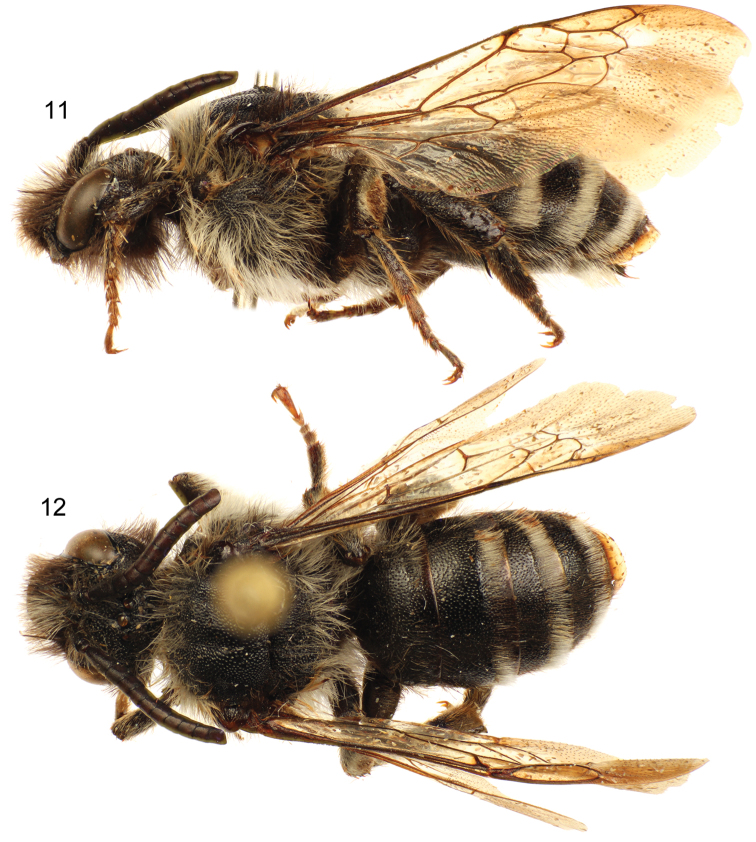
Lateral (**11**) and dorsal (**12**) habitus images of holotype male of *Sinomelecta oreina* Baker (SEMC Type No. 9280).

Figs 11 [Fig F6] [Fig F7] [Fig F8] –23

Sinomelecta oreina Baker, 1997: 246 [♂♀].

#### Holotype.

♂ (Figs 11–12), labeled “China: Szechuan, Ta-hsuëh Shan or Chiunghsia Shan” // “Holotype ♂, Sinomelecta oreina, D.B. Baker 1993 [actual publication date was 1997] [red label]”; deposited in the Division of Entomology, University of Kansas Natural History Museum, Lawrence, Kansas, USA.

#### Paratype.

1♀ (Figs 22–23), with same labels as holotype except second label blue and reading “Paratype ♀, Sinomelecta oreina, D.B. Baker 1993 [actual publication date was 1997] [blue label]”; deposited in the Division of Entomology, University of Kansas Natural History Museum, Lawrence, Kansas, USA.

#### Diagnosis.

As for the genus (*vide supra*).

#### Description.

*Male (holotype)*: Body length 11.5 mm (apex of metasoma damaged, as noted by Baker 1997, and dissected, so that body length measurement is not exact), forewing length 10 mm. Head narrower than thorax, head width 3.15 mm, head length (lower margin of clypeus to vertex in facial view) 2.75 mm. Intertegular distance 3.0 mm, distance between outer margins of tegulae 4.0 mm.

Clypeus strongly protuberant, in lateral view extending anteriorly more than compound eye width on front of compound eye margin; lower margin of clypeus straight, mandibles closed and difficult to see, but distal half narrow, tapering to narrowly rounded apex, no teeth visible. Malar space very short, base of mandible separated from compound eye by about one-fourth of distal diameter of F1. Labrum broader than long. Inner orbits weakly concave, slightly converging below (Fig. 20), vertex strongly convex as seen in facial view (Fig. 20); gena broadest near lower end, broader than compound eye in lateral view; preoccipital ridge rounded; ocelli about equal in diameter; ocellocular distance more than twice interocellar distance which is less than ocellar diameter; ocelloccipital distance about three ocellar diameters but indefinite because of rounding onto occiput. Distance between lateral and middle ocelli about two-thirds of ocellar diameter. Antenna with 12 antennomeres; scape (without basal bulb) nearly three times as long as its maximum width which is slightly less than apical width of F1; pedicel small, exposed part about three times as wide a long; F1 nearly twice as long as apical width and about twice as long as F2; F2 to F9 broader than long, F10 scarcely longer than F9, scarcely longer than broad, apex broadly rounded; flagellum on left hand side and as shown by Baker (1997: fig. 14) tapering slightly from F2 to F10 so that F2 maximum width is about 1.24 times F10 width, but on right hand side tapering less evident. Mesoscutellum with subhorizontal dorsal surface about twice as long as subvertical surface, both surfaces divided by weak longitudinal median depression so that mesoscutellum is weakly biconvex, each convexity emphasized by small posteriorly directed sublateral projection that almost overhangs metanotum. Metatibia with outer surface nodulose posteriorly; apex of protibia with two sharp spines, one anterior, the other posterior; other tibiae without evident apical spines although apical outer margin, especially of metatibia, protuberant; spurs of meso- and metatibiae about as long as tibial diameters, those of metatibia subequal in length; arolia small, much less than half as long as pretarsal claws; pretarsal claws cleft, inner ramus shaped more or less like outer ramus but somewhat shorter than outer ranus. Metasomal T1 with midlength of horizontal surface about half as long as declivitous anterior surface and about half as long as midlength of exposed part of T2, but since dorsal surface of T1 curves gradually onto anterior surface, these measurements are arbitrary; apical terga fragmented but apparent apex of T7 slightly less produced medially than S7 but notched like S7; surface of S7 with longitudinal median depression; posterior margins of S2 to S5 straight, transverse; S6 somewhat produced medially so that posterior margin is strongly convex; S7, S8, and genitalia shown in figures 15–19.

Setae mostly sparse enough that details of surface sculpturing visible (unlike in *Brachymelecta mucida*). Head including labrum coarsely and closely punctate, upper part of head somewhat more coarsely so than lower part; area in front of ocelli, extending down medially as frontal carina, smooth and impunctate; area around base of mandible, including malar space, smooth and shining; scape and basal half of mandible more finely punctate except for anterior apical smooth shining swelling of scape and smooth mandibular area near articulation. Mesosoma mostly coarsely and closely punctate, punctation similar to that of clypeus except somewhat coarser (like upper frons and vertex) in much of mesoscutum; median part of mesoscutum, except anteriorly, with many punctures separated from one another by smooth ground often one-third as wide as nearby punctures; lateral margin of mesoscutum (about as wide as nearby tegula), lateral extremity of mesoscutellum, and axilla much more finely punctate, these punctures as dense as they can be, variable but most about one-third as wide as those on disc of mesoscutum; posterior propodeal areas with sculpturing grading toward granular. Metasomal terga and sterna with coarse, dense, shallow punctures about size of punctures of frons; posterior margins of T1 to T3 narrowly impunctate, T4 more broadly impunctate, T5 with impunctate zone much broader than exposed punctate zone; S1 to S5 with narrow apical smooth margins.

Setae of head and mesosoma mostly four to five ocellar diameters in length, those of clypeus shorter than elsewhere; setae largely absent on posterior three-fourths of mesoscutum, mostly not branched, dull whitish or cinereous but dusky brownish on genal and hypostomal areas, grading to brownish cinereous on labrum and clypeus (although whitish on upper margin of clypeus medially) and black on lateral margin of clypeus, on lower paraocular area below level of antennal base, and on posterior lateral angle of mesoscutum, on upper part of axilla, and perhaps on extreme basolateral margin of mesoscutellum. Setae of antennal scape and basal parts of legs including upper surfaces of femora similar to cinereous setae of mesosoma but shorter; posterior margins of pro- and mesofemora with fringes of long, pale setae, slightly darkened apically on mesofemur; setae of flagellum and pedicel extremely short; under surfaces of femora with areas of brownish dusky setae, grading to dusky on outer surfaces of meso- and metatibiae; posterior outer surface of metatibia with large area of extremely dense, appressed setae that hide surface, these setae brown medially and whitish marginally; under surfaces of tarsi with short, yellowish brown setae. Metasomal T1 with long cinereous setae similar to those of mesosoma; T2 to T5 with similar setae, some of them brownish, at extreme sides; T2 to T4 with subapical bands of white setae (broken medially on T2); anterior to these bands setae inconspicuous, short (about one ocellar diameter in length), dusky; T5 largely hidden by T4, thus setal characters not clear but band of pale setae absent; S2 to S6 with long whitish setae, up to five ocellar diameters in length, sparse on S2 and S3, denser and forming preapical bands on S4 and S5.

Integumental coloration black throughout. Wings transparent, with brownish dusky stain in distal halves, darker distal to marginal cell, clear near and for short distance distal to 1rs-m and 2m-cu and along anterior margins of both submarginal cells; veins and pterostigma black.

*Female (paratype)*: As described for holotype male except as follows: Apex of metasoma intact. Forewing length 9.5 mm. Head width 3.3 mm; head length 2.8 mm; distance between outer margins of tegulae 4.25 mm.

Malar space a depressed groove, base of mandible separated from compound eye by about one-third of distal diameter of F1. Labrum broader than long, apical margin concave. Inner orbits more distinctly converging below than in male; ocellocular distance about 1.5 times interocellar distance, nearly three ocellar diameters; distance between lateral and median ocellus nearly one ocellar diameter. Apical width of F1 approximately equal to apical width of scape; pedicel fully exposed, broader than long; F1 about 2.5 times as long as its apical width, F2 about as long as broad, subsequent flagellomeres similar but progressively very slightly longer so that F7 is slightly longer than broad, F10 only slightly longer than F9 with apex broadly rounded; flagellum not tapering so that F1 and F10 approximately equal in width. Distitarsi broken off and lost except for one front leg that is difficult to see, although pretarsal claws smaller than in male; apical tibial spines represented by outer apical anterior and posterior protuberances except posterior spine of protibial apex present and that of mesotibia a large, long, blunt process; thickened spinelike setae also present on meso- and metatibial apices and on outer surface of metatibia; tibial spurs slightly longer than maximum tibial diameters. Metasomal T6 with well-defined pygidial plate, pointed at apex; T6 apparently produced as slender apical process above comparable slender apical process of S6 which has strongly notched apex exceeding tergal apex.

Area in front of ocelli not entirely smooth, with somewhat irregular punctures; scape without smooth, shining apical swelling. Metasomal terga and sterna with punctures slightly better spaced than in male, middorsally separated by shining ground one-third puncture width to full puncture width; apical impunctate margins of T1 to T3 slightly wider than in male, not contrasting with that of T4.

Most setae of legs from coxae to upper surfaces of tarsi whitish; area of dense setae on outer surface of mesotibia absent. Discal area of mesoscutum, upper inner extremity of axilla, and anterior margin of mesoscutellum with setae blackish (setae largely absent from discal area of mesoscutum in male). Metasomal T2 to T4 with discal setae largely palid and longer than in male so that subapical white setal bands contrast with their background less than in male; T5 and T6 without subapical bands of white setae; S2 to S5 with abundant long whitish setae, those of S2 and S3 not contrastingly sparse, as in male.

**Figures 13–14. F6:**
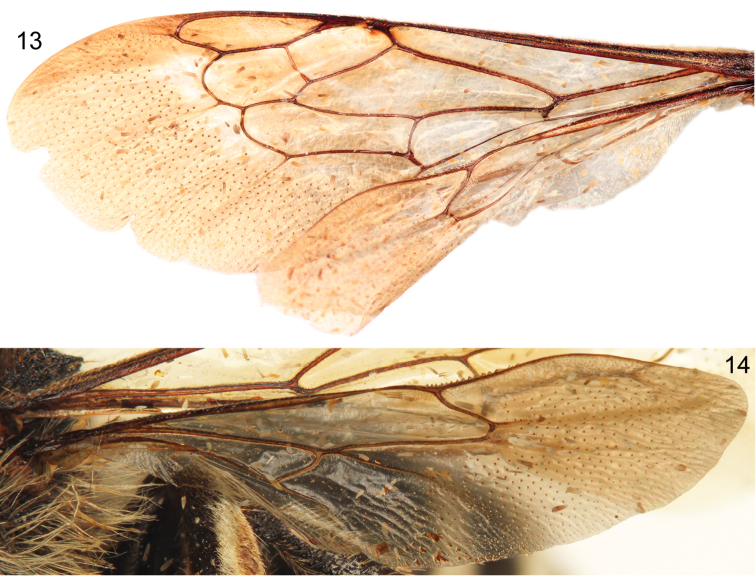
Forewing (**13**) and hind wing (**14**) of holotype male of *Sinomelecta oreina* Baker (SEMC Type No. 9280).

**Figures 15–19. F7:**
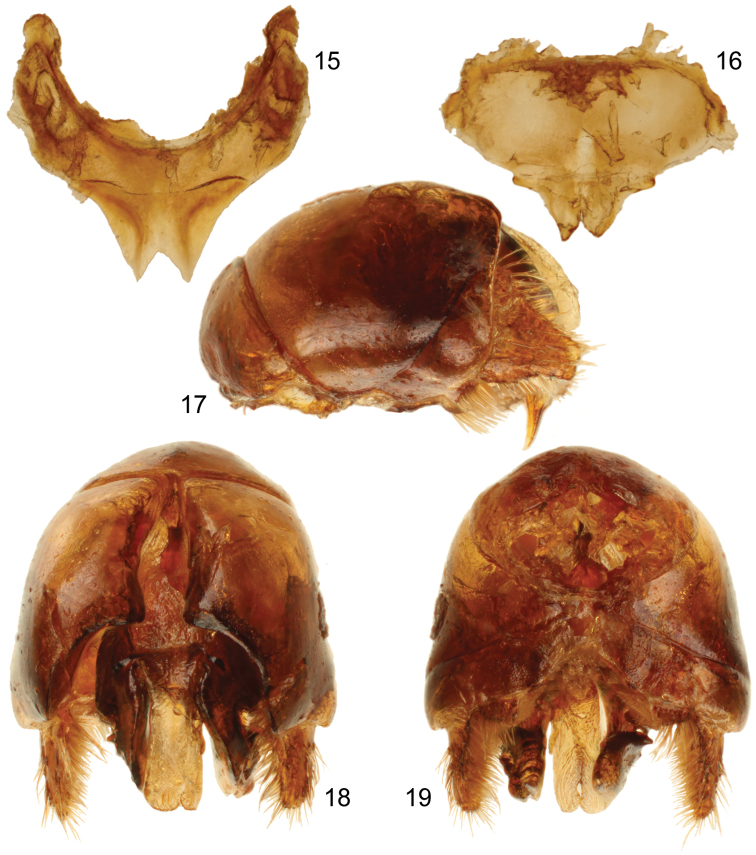
Male terminalia of holotype of *Sinomelecta oreina* Baker (SEMC Type No. 9280). **15** Seventh metasomal sternum **16** Eighth metasomal sternum **17** Genital capsule, lateral view **18** Genital capsule, dorsal view **19** Genital capsule, ventral view.

**Figures 20–21. F8:**
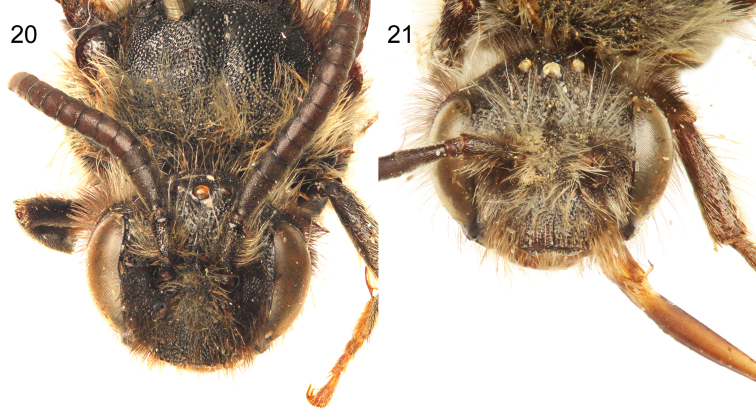
Male holotype (**20**) and female paratype (**21**) facial views of *Sinomelecta oreina* Baker (SEMC Type No. 9280).

**Figures 22–23.**
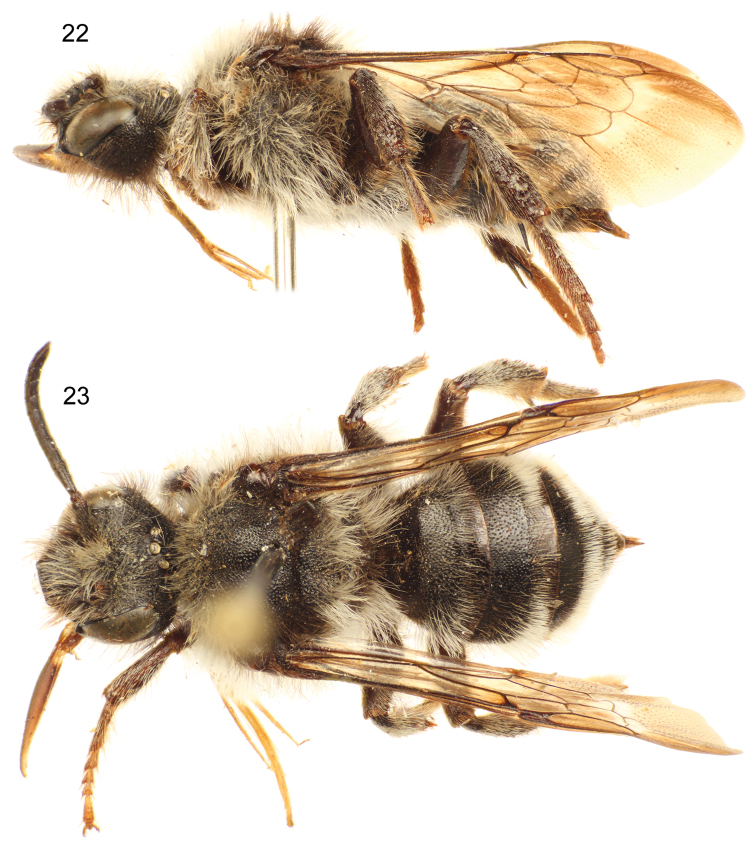
Lateral (**22**) and dorsal (**23**) habitus images of paratype female of *Sinomelecta oreina* Baker (SEMC Type No. 9280).

#### Comments.

Although peculiar, the 12 antennomeres in the male of *Sinomelecta oreina* is not unheard of among bees and this condition is found in various genera. For example, this same reduced antennomere count is well known and fixed across species in the augochlorine genus *Chlerogas* Vachal (Brooks and Engel 1999; Engel 2000), the ammobatine genus *Chiasmognathus* Engel (Engel 2006, 2009), the biastine genus *Neopasites* Ashmead (Linsley 1943), and the ammobatoidine genus *Holcopasites* Ashmead (Hurd and Linsley 1972; Michener 2007). Accordingly we do not consider this to merely represent an isolated teratology.

Note that the mention of “*Melecta oreina* Baker” in the key to Eastern Hemisphere genera of Melectini by Rightmyer and Engel (2003) should have read “*Melecta emodi* Baker” (*oreina* is, of course, the type species of *Sinomelecta* which they recognized as a valid genus).

## Discussion

*Brachymelecta mucida* is wholly unique in the North American fauna. Not only is it completely dissimilar from the other known melectine fauna for the continent, superficially more similar to some Asiatic forms, but also the type specimen is the only known individual to have ever been collected, despite considerable effort in Nevada and neighboring states. It is even possible that the species is today extinct, although not too much can be made from decades of absence data as putatively lost species can still turn up [e.g., *Epeoloides pilosula* (Cresson) which was not seen for 42 years before being recaptured in Nova Scotia and Connecticut (Sheffield et al. 2004; Wagner and Ascher 2008)]. Nonetheless, the prospects of recovering new material, as well as the unknown female, certainly dwindle with each passing year. Naturally, this complete absence of additional or new material leads one to speculate whether *Brachymelecta mucida* is even native to North America, its association with Morrison’s material being erroneous, potentially leaving the specimen mistakenly labeled as coming from “Nev.” The currently available historical evidence certainly does not support such a conjecture as no clearly foreign samples of Hymenoptera appear to have been received and processed in Philadelphia at about the same time as the material from Morrison’s 1878 field work in Nevada (Mann 1885). Although Morrison did sell material to other institutions and collectors (e.g., Horn et al. 1990; Rasmussen 2012), all specimens were collected in the USA and therefore unlikely to have been mixed with material from foreign sources (at least not mixed by Morrison!). Thus, in the absence of clear, or even mildly suggestive, historical documents to imply otherwise, we consider *Brachymelecta mucida* native to the western United States, presumably to an undisclosed region in Nevada. It is, without a doubt, the most rare of North American bee species, assuming it is still living.

In regard to *Sinomelecta*, it may be tantalizing to suspect that *Sinomelecta oreina*, with its various external features reminiscent of Palearctic *Melecta* [e.g., *Melecta (Melecta) emodi* Baker], is merely an autapomorphic species of that genus with its two submarginal cells and reduced flagellar count in males. This might seem particularly likely given that 1rs-m is lost easily in apoids, thereby resulting in the two submarginal cell condition. From Baker’s (1997) account and figures there was no reason to readily exclude a placement within *Melecta*, perhaps considering the taxon as a subgenus. Indeed, the genital capsule is somewhat reminiscent of *Pseudomelecta* Radoszkowski in its broad, squat form with a broad gonostylus that is densely setose throughout, and with a broad ventral setose lobe (*vide* Lieftinck 1972). The hidden sterna, however, are quite unlike those of *Pseudomelecta*. Indeed, the seventh sternum is rather different from that of *Melecta*, more closely resembling the form found in *Thyreus* [e.g., *Thyreus ramosus* (Lepeletier de Saint Fargeau): *vide* Lieftinck 1968: figs 31–33]. In addition, the long first flagellomere and narrowly triangular pygidial plate of the female are unlike *Melecta* and the form of the mesoscutellum, pubescence, and presence of arolia readily exclude *Sinomelecta oreina* from *Thyreus*. Naturally, cladistic work is needed throughout the Melectini, particularly to determine whether *Melecta* as constituted is monophyletic, and it is hoped that this fuller account of two rare genera might encourage and bolster such work.

## Supplementary Material

XML Treatment for Brachymelecta

XML Treatment for Brachymelecta mucida

XML Treatment for Sinomelecta

XML Treatment for Sinomelecta oreina
